# Bidirectional and dynamic relationships between social isolation and activities of daily living among older adults in China

**DOI:** 10.7189/jogh.14.04031

**Published:** 2024-01-26

**Authors:** Chaoping Pan, Linwei Yu

**Affiliations:** College of Medical Humanities and Management, Wenzhou Medical University, Wenzhou City, Zhejiang Province, China

## Abstract

**Background:**

Social isolation and disability in performing activities of daily living are increasingly recognised as significant public health concerns globally. We aimed to investigate their bidirectional associations and the related temporal dynamics in Chinese older adults.

**Methods:**

We retrieved data from the six waves of the Chinese Longitudinal Healthy Longevity Survey (2002–18) and used generalised cross-lagged modelling (GCLM) to assess the bidirectional associations between social isolation and disability in performing activities of daily living.

**Results:**

We found that higher levels of social isolation were predictive of increased scores in disabilities in performing activities of daily living. Conversely, disabilities in performing activities of daily living showed less predictive power in relation to social isolation. The temporal dynamics analysis indicated a peak in the bidirectional associations after approximately six years, followed by decreasing trends.

**Conclusions:**

Our results indicate that social isolation is dominant in the bidirectional relationship. Efforts focusing on reducing it can potentially minimise disabilities in performing activities of daily living among older adults. Reinstating preventive interventions beyond the six-year mark could help maintain their effectiveness.

Population ageing worldwide has led to an increased prevalence of disabilities among older individuals, which can lead to significant health care expenses, hospitalisations, future falls, injuries, cognitive decline, and even mortality [1-3]. Assessing activities of daily living (ADL) is crucial, as it measures an individual's functional capacities necessary for essential daily tasks like bathing, dressing, and toileting [4]. Disability in performing ADL is a more severe limitation that poses a greater threat to older adults’ well-being and lives compared to other forms of disability, such as instrumental ADL and functional limitation [4,5]. It is highly prevalent among Chinese older adults, affecting almost 10% of the population [6,7]. Given these severe consequences and high prevalence, there is a need for identifying key risk factors and developing effective interventions to address disability in performing ADL in this population.

Social isolation (SI) is commonly described as having limited social contact, minimal engagement in social activities, and living alone [8]. Research has consistently associated it with various adverse health outcomes, including disability, cognitive decline, and mortality [9-11]. With the global population ageing at a rapid pace, SI has emerged as a significant concern among older adults, with studies indicating that 10%–43% of older adults experience it in later stages of life [12], with a particularly high prevalence of 42.4% reported among older adults in China [13]. These findings underscore the importance of implementing strategies aimed at preventing or reducing the development of SI among older adults.

A growing body of literature suggests that SI and ADL disability are not separate phenomena, but rather interconnected. One line of research has explored a unidirectional relationship where SI is considered an explanatory factor for disability in performing ADL [9,14]. For example, Guo et al. [14] found that higher levels of SI contribute to increased disability in performing ADL, but exclusively among older adult women. Although the specific mechanisms driving this relationship have yet to be extensively studied, potential factors may include health behaviours, stress levels, and the ability to engage in repair and maintenance activities [15]. Conversely, others have suggested a unidirectional association where ADL disability serves as the explanatory variable for SI outcomes. These studies indicated that disability in performing ADL can lead to increased SI among older adults and have documented the effects of health interventions on reducing SI [16,17]. This association may stem from the limitations imposed by disability in performing ADL, hindering older adults from engaging in social activities, visiting friends or relatives, and participating in physical activities [18]. Therefore, it is plausible that the relationship between SI and disability in performing ADL is, in fact, bidirectional.

To the best of our knowledge, no studies have yet used appropriate statistical methods to investigate the bidirectional relationship between SI and disability in performing ADL. Certain challenges need to be addressed to establish this relationship accurately. First, current estimates regarding the impact of disability in performing ADL on SI and vice versa may suffer from spurious connections resulting from reverse causality [8,19,20]. This makes it necessary to employ methods that account for reverse causality to determine the causal ordering between these two processes and obtain accurate estimates of their associations. Second, it remains unclear which factor has a more influential effect, which has implications for targeting preventative measures effectively. This means that interventions should be focused on whichever phenomenon has a greater influence in the bidirectional relationship [16,18]. Lastly, failing to consider the bidirectional relationship between SI and disability in performing ADL can hinder our understanding of the temporal dynamics between these factors. The time frame for the dose-response relationship between SI and disability in performing ADL, as well as the autoregressive properties of these phenomena, are currently not known. Enhancing our comprehension of these dynamics will optimize the timing of interventions aimed at reducing SI and disability in performing ADL among older adults, increasing their efficiency and effectiveness [21].

To address these challenges, we aimed to examine the bidirectional associations between SI and disability in performing ADL among older adults and to established the temporal dynamics and effect horizons associated with these associations. To achieve this, we used 17 years of follow-up data from the Chinese Longitudinal Healthy Longevity Survey (CLHLS), a nationally representative community-based survey conducted in China, specifically focusing on older adults. We employed a general cross-lagged panel model (GCLM) to analyse the data and investigate the bidirectional relationships between SI and disability in performing ADL.

## METHODS

### Data and participants

The CLHLS was conducted in 23 out of the 31 provinces in China and covered approximately 85% of the population. The baseline survey was conducted in 1998, with multiple subsequent waves in 2000, 2002, 2005, 2008, 2011, 2014, and 2018 [22,23]. It began including individuals aged ≥65 years in 2002; we therefore used the data the waves from this year onwards (inclusive of 2002). We excluded individuals aged <65 years across all waves (as they represented only 1% of the total sample) and those who were tracked less than twice (due to the longitudinal nature of the study). The sample distribution by years was as follows: 2002 (n = 8136), 2005 (n = 11 427), 2008 (n = 11 571), 2011 (n = 9194), 2014 (n = 6551), and 2018 (n = 3469).

Notably, we had missing data of 1.6% and 1.9% for SI and ADL, respectively. We resolved this through multiple imputation, an efficient method to address the potential attrition bias due to missing values in longitudinal analysis [24]. Specifically, we used the Markovchain Monte Carlo method with five imputations, which was previously deemed sufficient to generate robust estimates through multiple imputations [24]. Additional information regarding the sample characteristics and correlations of the analytic variables can be found in Tables S1–2 in the **Online Supplementary Document**.

### Measures

#### ADL

The measurement of performing ADL consisted of six items: bathing, dressing, toileting, indoor transferring, continence, and feeding. The items for assessing level of disability in performing ADL were scored on scale ranging from 0 to 3: no limitation (scored as 0), a little difficulty (scored as 1), and unable to perform the task (scored as 2). The total ADL score thus ranged from 0 to 12, with higher scores indicating a higher level of disability in performing ADL.

#### SI

Based on recommendations from previous literature [2,13,14,25,26], we used five dimensions to capture different aspects of SI: living alone, having a spouse, frequent contact with children, frequent contact with siblings, and participation in social activities. In CLHLS, participants were directly asked about their living arrangements, frequency of contact with children and siblings, and their involvement in social activities such as organised social activities and playing cards or mahjong. One point was given if participants lived alone, lacked a spouse, were infrequently visited by children/siblings, or had limited social participation. The total SI scores ranged from 0 to 5, with higher scores indicating a higher level of SI.

### Analysis

We used GCLM to analyse the data, following a method proposed by Zyphur et al. [21]. This approach allows for estimating the bidirectional associations between two variables and visualising the temporal dynamics in their relationships. It is increasingly used in research examining the bidirectional connections between social aspects and health outcomes, such as SI and physical functioning [27]. To fit the model, we used MPlus 8 software (Muthén & Muthén, Los Angeles, CA, USA) within a structural equation modelling framework. The model specification is:

*ADL_it_* = β_1_*SI_it_* _− 1_ + β_2_*ADL_it_* _− 1_ + *θ_t_* + *μ_i_* + *ε_it_*

*SI_it_* = γ_1_*ADL_it_* _− 1_ + γ_2_*SI_it_* _− 1_ + *θ_t_* + *α_i_* + *e_it_*

where *_i_* and *_t_* represent individuals and time periods, respectively; *SI* represents social isolation; and *ADL* denotes disability in performing ADL. The regression coefficients β_1_, β_2_, γ_1_, and γ _2_ are estimated from the model. θ and σ represent occasion effect, μ and α capture the time-invariant effect, and ε and *e* represent the idiosyncratic error terms. Importantly, the model implicitly accounts for time-varying and time-invariant confounders through the inclusion of correlation terms between ε and *e*, as well as μ and α [21]. The cross-lagged coefficients β_1_ and γ_1_ are of key interest as they indicate whether and how differences in SI and ADL disability at a given time point predict differences in ADL disability and SI at the next time point, respectively. The autoregressive paths β_2_ and γ_2_ reflect the extent to which within-individual deviations from expected scores in ADL and SI, respectively, can be predicted from deviations from their past scores. The model also allows for the calculation of the time horizon of dose-response relationships between ADL and SI, as well as the autoregressive properties through linear combinations of the regression coefficients [21]. Prior to inclusion in the model, we standardised the variables to facilitate comparisons across different variables used in the analysis. We then conducted nonparametric bootstrapping with 10000 replications to calculate confidence intervals (CIs), following a previously suggested approach [27]. This technique is a resampling method that does not assume an underlying parametric distribution, making it a more flexible and robust approach when dealing with non-normal data. We assessed multiple model fit indices to confirm the goodness of model fit, including the confirmatory fit index, the Tucker Lewis index, the root mean square error of approximation, and the standardised root mean squared residual [21].

## RESULTS

The study participants had a mean age of 81.83 years; 54.8% were female. Approximately 42.5% of the participants had received at least one year of education, while most (56.1%) resided in rural regions. The participants had an average ADL score of 0.46 and average SI score of 2.87 (Table S1 in the **Online Supplementary Document**).

### The bidirectional associations between SI and disability in performing ADL

Our findings indicate that higher levels of SI at a given time point were associated with an increased risk of disability in performing ADL in the future, as indicated by the significant coefficient β_1_. Specifically, for each increment of one SD in SI, there was an average increase of 0.022 SDs in disability in performing ADL (95% CI = 0.006, 0.071). The analysis did not reveal a significant association between disability in performing ADL at a given time point and SI in the future (γ_1_ = 0.002; 95% CI = −0.015, 0.037). Additionally, the autoregressive coefficients for ADL disability (β_2_ = 0.196; 95% CI = 0.147, 0.318) and SI (γ_2_ = 0.302; 95% CI = 0.265, 0.370) affirmed that past disability in performing ADL and SI could influence their future levels (**Table 1**).

**Table 1:** Key model parameters and selected goodness-of-fit statistics from GCLM (CLHLS, waves 2002–18)

	β (95% CI)
**Model parameters**	
SI_t −1_ → SI_t_	0.302 (0.265, 0.370)
SI_t −1_ → ADL_t_	0.022 (0.006, 0.071)
ADL_t −1_ → ADL_t_	0.196 (0.147, 0.318)
ADL_t −1_ → SI_t_	0.002 (−0.015, 0.037)
**Goodness-of-fit statistics**	
CFI	0.990
TLI	0.984
RMSEA	0.020
SRMR	0.033

ADL – activities of daily living, CFI – confirmatory fit index, RMSEA – root mean square error of approximation, SI – social interaction, SRMR – standardised root mean squared residual, TLI – Tucker Lewis index

### The dynamic relationships between SI and ADL disability

**Figure 1:**
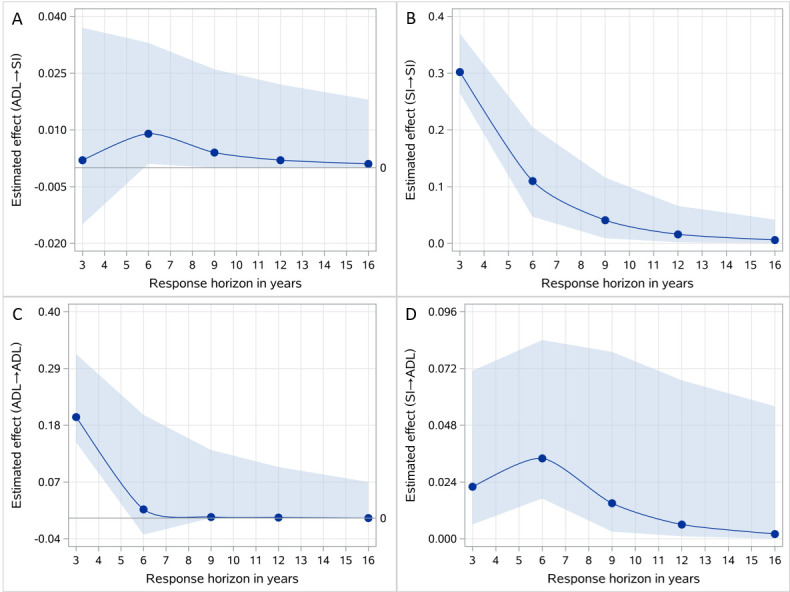
Effect of past within-subject variations in SI and ADL in the year 2002 on future scores (CLHLS, waves 2002–18).

Regarding the dynamic influence of past within-subject variations in SI and disability in performing ADL on future scores based on 2002 as the baseline year, the cross-lagged effects demonstrated an initial increase, reaching their highest points at six years, then gradually declining. Conversely, the influence of disability in performing ADL on SI seemed to be significant only at the six-year mark. Furthermore, both the autoregressive paths for SI and disability in performing ADL exhibited decreasing trends over time (**Figure 1**, Panels A–C).

### Sensitive analysis

To ensure the robustness of our results using multiple imputation methods, we conducted sensitivity analyses by removing missing data and re-estimating the bidirectional associations. We found consistent results compared to the main analyses, thus confirming the robustness of our findings (Table S3 in the **Online Supplementary Document**).

## DISCUSSION

We derived significant insights from the analysis of nationally representative data using the GCLM. We found that SI and disability in performing ADL influence each other reciprocally, with observed temporal trends. This bears considerable significance for the design of preventive strategies aimed at fostering health and well-being within this demographic.

The first finding of this study highlights that higher levels of SI at a given time point are associated with increased scores in disability in performing ADL, whose impact on SI, in turn, bears less significance. The influence of SI on disability in performing ADL may occur through three pathways: health behaviour, stress, and repair and maintenance [15]. Meanwhile, limitations in ADL may result in older adults being less engaged in social activities, visiting friends/relatives, and participating in physical activities, thereby contributing to the effect of disability in performing ADL on SI [18]. These findings align with previous studies that reported unidirectional associations between SI and disability in performing ADL [8,19,20] by confirming the interrelatedness of these two processes in complex ways that traditional models fail to fully capture. Overall, our findings suggest that the effect of SI on ADL may hold greater prominence within the bidirectional relationship between these variables.

A second key finding of the study indicated that the temporal dynamics within the bidirectional relationships between SI and disability in performing ADL were both significant, with a peak after six years followed by decreasing trends. However, these findings differ from a previous study, which only identified decreasing trends that gradually lost significance in the temporal dynamics of the bidirectional relationships between SI and functional limitation among older adults [27]. This dissimilarity could be related to the use of different disability indicators. While we specifically focused on ADL disability, the aforementioned study investigated functional limitation [27], which may have a significantly higher incidence compared to disability in performing ADL [5]; this disparity tends to widen as older adults age. This difference in prevalence might explain why the effect of SI on functional limitation did not reach statistical significance in later years in that study [27].

Our findings have significant implications for interventions targeting the health and well-being of older adults in China and similar countries. First, they highlight that SI has a more pronounced effect on disability in performing ADL than vice versa. Consequently, policy strategies aimed at improving the health outcomes of older adults should prioritise reducing SI to enhance intervention efficiency and effectiveness. In this way, not only can its prevalence be reduced, but it can also contribute to a reduction in disability in performing ADL, ultimately leading to improved overall well-being among older populations. Second, it is important to recognise the temporal dynamics observed in the bidirectional effects, with their peak occurring after approximately six years. This suggests that the effectiveness of interventions targeting SI and disability in performing ADL may gradually diminish over time following the six-year mark. Consequently, it becomes imperative to reissue relevant interventions to improve their effectiveness beyond this timeframe.

This study has multiple strengths. It is based on a large, nationally representative sample of individuals aged ≥65 years, ensuring that the findings can be generalised to the broader population of Chinese older adults. Second, we employed a novel GCLM that effectively addresses potential confounding factors, including reverse causality. By considering both observable and unobservable time-invariant and time-varying confounds, this model strengthens the ability to draw causal inferences from the data. Additionally, the GCLM enables the identification of temporal dynamics in the interrelationships between SI and ADL disability, providing valuable insights into the nature of these relationships over time. Finally, we adopted a well-validated objective measure of disability in performing ADL, which may improve the accuracy and reliability of our findings.

However, this study also has some limitations. For example, the measurement of SI did not include contacts with non-family members. To enhance the generalisability of our findings, future research could replicate our study and incorporate non-family members, such as friends and neighbours, into the measurement of SI. Another limitation is that we solely relied on objective measures of social relationships. Future research could delve deeper into the interplay between subjective social relationships, such as feelings of loneliness, and the concept of disability in performing ADL among older adults.

## CONCLUSIONS

The study findings suggest that SI may play a more prominent role than disability in performing ADL within their bidirectional relationship. This highlights a need for targeted interventions that effectively address SI to promote better health outcomes and overall well-being among older adults. Moreover, the analysis of the effects’ time horizons offers practical insights for determining the most effective timing to deliver preventive interventions, allowing for optimal impact on reducing SI and preventing disability in performing ADL among older adults.

## Additional material


Online Supplementary Document
