# Supplementary material for: Bidirectional and dynamic relationships between social isolation and activities of daily living among older adults in China
Source: J Glob Health. 2024 Jan 26;14:04031. doi: 10.7189/jogh.14.04031 (PMC10811563; doi:10.7189/jogh.14.04031)
Supplement: Online Supplementary Document [file jogh-14-04031-s001.pdf]

Supplemental materials

Table S1 baseline characteristics of sample in 2002

Variable	Mean (SD) or %
Social isolation	2.87 (1.01)
ADL	0.46 (1.42)
Age	81.83(10.87)
Sex, female (%)	54.8
Education, received at least one year of education (%)	42.5
Residence, rural (%)	56.1

Table S2 Bivariate Correlations Between ADL disability and SI

	ADL2	ADL3	ADL4	ADL5	ADL6	SI1	SI2	SI3	SI4	SI5	SI6
ADL1	0.378	0.252	0.237	0.171	0.119	0.167	0.154	0.122	0.098	0.085	0.011
ADL2	1	0.360	0.269	0.214	0.058	0.183	0.216	0.149	0.094	0.066	0.042
ADL3		1	0.389	0.214	0.065	0.159	0.194	0.193	0.143	0.085	0.040
ADL4			1	0.492	0.155	0.145	0.177	0.214	0.220	0.165	0.074
ADL5				1	0.288	0.105	0.140	0.193	0.191	0.206	0.105
ADL6					1	0.122	0.101	0.169	0.173	0.165	0.231
SI1						1	0.613	0.553	0.515	0.409	0.276
SI2							1	0.623	0.575	0.471	0.326
SI3								1	0.774	0.525	0.412
SI4									1	0.600	0.500
SI5										1	0.567

Table S3 Key model parameters and selected goodness-of-fit statistics from GCLM in sensitive analysis (CLHLS, Waves 2002–2018)

	Standardized Coefficient (95% CI)
Model parameters	
$SI_{t-1} \rightarrow SI_t$	0.305 (0.262, 0.393)

$SI_{t-1} \rightarrow ADL_t$	0.023 (0.003, 0.064)
$ADL_{t-1} \rightarrow ADL_t$	0.200 (0.144, 0.278)
$ADL_{t-1} \rightarrow SI_t$	0.001 (-0.015, 0.031)
Goodness-of-fit statistics	
CFI	0.990
TLI	0.984
RMSEA	0.020
SRMR	0.039

Notes: CFI = Confirmatory Fit Index; TLI = Tucker Lewis Index; RMSEA = Root Mean Square Error of Approximation; SRMR = Standardized Root Mean Squared Residual.
